# Deletion of the LTR Enhancer/Promoter Has No Impact on the Integration Profile of MLV Vectors in Human Hematopoietic Progenitors

**DOI:** 10.1371/journal.pone.0055721

**Published:** 2013-01-31

**Authors:** Arianna Moiani, Annarita Miccio, Ermanno Rizzi, Marco Severgnini, Danilo Pellin, Julia Debora Suerth, Christopher Baum, Gianluca De Bellis, Fulvio Mavilio

**Affiliations:** 1 Division of Genetics and Cell Biology, Istituto Scientifico H. San Raffaele, Milan, Italy; 2 Department of Life Sciences, University of Modena and Reggio Emilia, Modena, Italy; 3 Institute for Biomedical Technologies, Consiglio Nazionale delle Ricerche, Milan, Italy; 4 CUSSB, Vita-Salute San Raffaele University, Milan, Italy; 5 Institute of Experimental Hematology, Hannover Medical School, Hannover, Germany; 6 Genethon, Evry, France; Mayo Clinic, United States of America

## Abstract

Moloney murine leukemia virus (MLV)-derived gamma-retroviral vectors integrate preferentially near transcriptional regulatory regions in the human genome, and are associated with a significant risk of insertional gene deregulation. Self-inactivating (SIN) vectors carry a deletion of the U3 enhancer and promoter in the long terminal repeat (LTR), and show reduced genotoxicity in pre-clinical assays. We report a high-definition analysis of the integration preferences of a SIN MLV vector compared to a wild-type-LTR MLV vector in the genome of CD34^+^ human hematopoietic stem/progenitor cells (HSPCs). We sequenced 13,011 unique SIN-MLV integration sites and compared them to 32,574 previously generated MLV sites in human HSPCs. The SIN-MLV vector recapitulates the integration pattern observed for MLV, with the characteristic clustering of integrations around enhancer and promoter regions associated to H3K4me3 and H3K4me1 histone modifications, specialized chromatin configurations (presence of the H2A.Z histone variant) and binding of RNA Pol II. SIN-MLV and MLV integration clusters and hot spots overlap in most cases and are generated at a comparable frequency, indicating that the reduced genotoxicity of SIN-MLV vectors in hematopoietic cells is not due to a modified integration profile.

## Introduction

Retroviral integration is a non-random process, whereby the viral RNA genome, reverse transcribed into double-stranded DNA and assembled in pre-integration complexes (PICs), associates with the host cell chromatin and integrates through the activity of the viral integrase [1]. Large-scale surveys of retroviral integration in murine and human cells uncovered some genomic features systematically and specifically associated with retroviral insertions, and revealed that each retrovirus type has a unique, characteristic pattern of integration within mammalian genomes [2]. Target site selection depends on both viral and cellular determinants, poorly defined for most retroviruses. The Moloney murine leukemia virus (MLV) and its derived vectors integrate preferentially in transcriptionally active promoters and regulatory regions [2], [3], [4], while the simian (SIV) and human immunodeficiency virus (HIV) and their derived lentiviral vectors target gene-dense regions and the transcribed portion of expressed genes, away from regulatory elements [2], [4], [5].

Recent clinical studies have shown that transplantation of stem cells genetically modified by retroviral vectors may cure severe genetic diseases such as immunodeficiencies [6], [7], [8], skin adhesion defects [9] and lysosomal storage disorders [10]. Some of these studies showed also the genotoxic consequences of retroviral gene transfer technology. Insertional activation of proto-oncogenes by MLV-derived vectors caused T-cell lymphoproliferative disorders in patients undergoing gene therapy for X-linked severe combined immunodeficiency [11], [12] and Wiskott-Aldrich syndrome [13], and pre-malignant expansion of myeloid progenitors in patients treated for chronic granulomatous disease (CGD) [14], [15]. The strong transcriptional enhancers present in the MLV LTR played a major role in deregulating gene expression. Pre-clinical studies showed that enhancer-less, self-inactivating (SIN) MLV-derived vectors are less prone to insertional oncogenesis and cell immortalization than their full-LTR counterparts, with a genotoxic profile closer to that of SIN-HIV vectors [16], [17], [18], [19]. The MLV U3 enhancer contains repeated binding sites for cellular transcription factors (TF), which may play a role in tethering retroviral pre-integration complexes to transcriptionally active regulatory regions and contribute to the MLV genotoxic characteristics [20].

In this study, we have analyzed the integration profiles of a MLV and SIN MLV vectors in the genome of a clinically relevant target cell population, cord blood-derived CD34^+^ hematopoietic stem/progenitor cells (HSPCs), by ligation-mediated PCR (LM-PCR) and high-throughput sequencing. We show that SIN-MLV and MLV vectors have very similar integration preferences, with the typical clustering around enhancer and promoter regions associated to specific histone modifications, specialized chromatin configurations and binding of RNA Pol II. Strikingly, SIN-MLV and MLV integration clusters and hot spots overlap in most cases and are generated at a similar frequency, indicating that the U3 enhancer has no role in targeting MLV PICs to the genome, at least in hematopoietic cells.

## Results

### MLV and SIN-MLV vectors share the same integration profile in the genome of human HSPCs

To generate a high-definition integration profile of SIN MLV integrations in human HSPCs, we transduced umbilical cord blood-derived CD34^+^ cells with a previously described SIN-MLV vector carrying a GFP expression cassette under the control of the human elongation factor 1α (EFS) promoter [16], [21]. Cells were transduced at 40 to 60% efficiency, and were selected for GFP expression by cell sorting 10 days after infection, to dilute unintegrated vectors. Vector-genome junctions were amplified from genomic DNA by ligation-mediated (LM)-PCR and pyrosequenced as previously described [4]. Raw sequences (available at the NCBI Sequence Read Archive with the accession number SRA061405) were processed by a previously described bioinformatic pipeline [4] and mapped on the University of California at Santa Cruz (UCSC) hg19 release of the human genome (http://genome.ucsc.edu), to obtain 13,011 unique insertion sites. Two previously generated datasets of full-LTR MLV vector integrations (32,574) and random sites (40,000) normalized for a number of parameters [4] were re-annotated on the hg19 release of the human genome and used for comparison.

**Figure 1:**
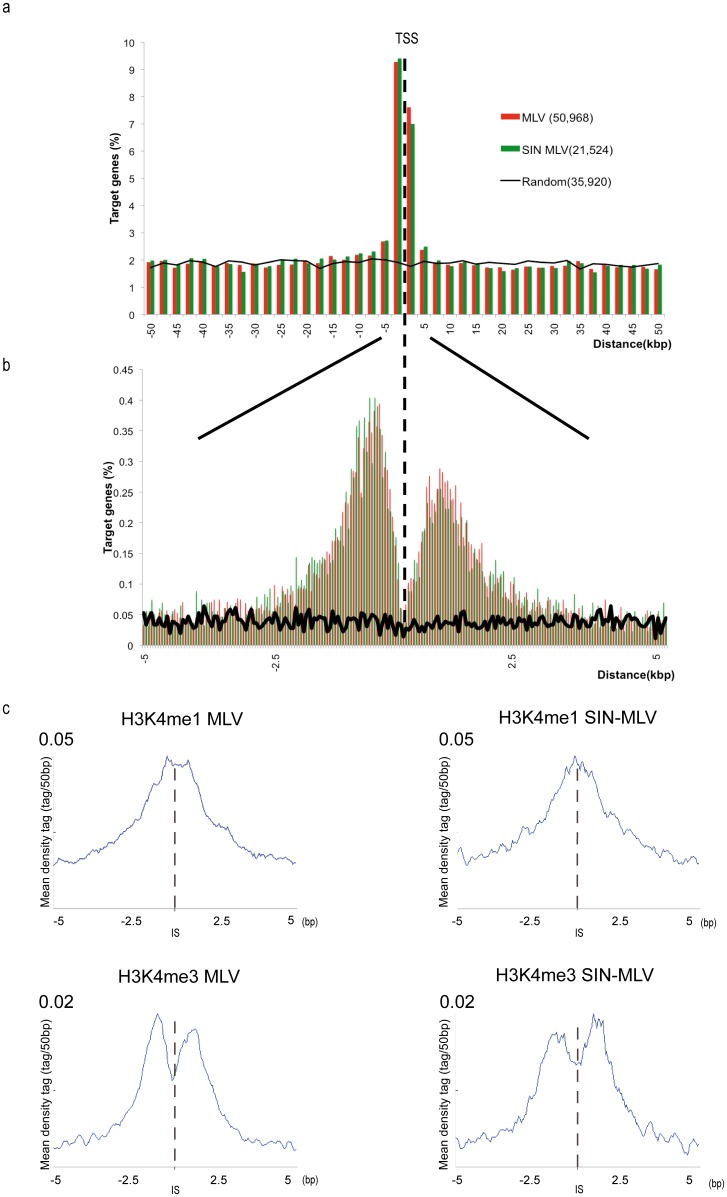
Genomic distribution and association with histone modifications of MLV and SIN-MLV integrations in human HSPCs. Distribution of the distance of SIN-MLV (green bars) and MLV (red bars) integration sites from the TSS of targeted genes at 2,500 bp (**a**) or 50 bp (**b**) resolution. The percentage of genes targeted at each position is plotted on the y axis. The number of plotted sites is higher than the actual number of mapped integrations sites, since each site may relate to more than one gene. The black line indicates the distribution of random control sites. (**c**) The distribution of H3K4me1 (top panels) and H3K4me3 (lower panels) epigenetic marks in a 10-kb window around vector integration sites (IS) is shown for MLV integrations (left panels) or SIN-MLV integrations (right panels). The mean density of tags (tag/50 bp) of the reference dataset (i.e. each chromatin feature) is plotted on the y axis. The scale of the graph is shown at the top left of each panel.

To identify differences in the integration preferences of MLV and SIN-MLV in HSPCs, we first analyzed the relationship between integration sites and Known Genes (UCSC definition) in the human genome: integration were annotated as TSS-proximal when occurring in an interval of ±2.5 kb from the TSS of any Known Gene, intragenic when occurring inside a Known Gene >2.5 kb from the TSS, and intergenic in all other cases. Intergenic and intragenic integrations were <40% for both MLV and SIN-MLV, while TSS-proximal integrations were 22.9% and 23.8% respectively (*P*>0.1 for all comparisons) (**Table 1**). Plotting the relative distance of all integration sites in an interval of ±50 kb from any TSS showed two virtually overlapping distributions with the characteristic enrichment in the ±2.5 kb interval and, at 50-bp resolution, the characteristic drop in frequency in close proximity (±0.2 kb) of a TSS (**Figure 1**). Similarly, integrations were enriched around annotated CpG islands (UCSC track) for both MLV and SIN-MLV vectors (12.8 and 12.4% respectively, *P*>0.1), and showed again overlapping, bimodal distributions in the ±2.5 kb interval around the island midpoint (**Figure S1A**). Finally, the moderate enrichment in the frequency of integration around mammalian, evolutionarily conserved non-coding sequences (CNC) [22] was the same for both vectors (8.0 and 7.7% respectively, *P*>0.1), and showed a virtually identical distribution at ±2.5 kb around the feature midpoint (**Figure S1B**). In all cases, there were significant differences between the distributions of the two MLV vectors and that of the random controls (*P*<10^−15^).

**Table 1:** Distribution of SIN-MLV and MLV integrations in the genome of human HSPCs.

	Intergenic (%)	TSS-proximal (%)	Intragenic (%)	CpG islands (%)	CNCs (%)	Total hits
**SIN-MLV**	38.0	23.8	38.1	12.4	7.7	13,011
**MLV**	38.2	22.9	38.8	12.8	8.0	32,574
**Random**	59.1	3.0	37.8	1.2	5.4	40,000

Percentage of intergenic, TSS-proximal or intragenic integration sites in the SIN-MLV, MLV and random control site datasets, together with the percentage of sites at a distance of ±1,000 bp from at least one CpG island or conserved non coding region (CNC).

We previously reported that MLV integrations are strongly associated with histone modifications marking transcriptionally active promoters and enhancers, with the specialized H2A.Z histone variant, and with binding sites for RNA Pol II and transcription factors in both HSCs and T cells [4], [20], [23]. Taking advantage of published ChIP-Seq datasets on epigenetic features in the chromatin of human CD34^+^ HSPCs [24], we analyzed the association of MLV and SIN-MLV integrations with histone modifications marking active enhancers and promoters (H3K4me1, H3K4me3) and heterochromatin (H3K27me3), H2A.Z and binding of Pol II. In all cases, there was no obvious difference between the two vectors: we observed a strong association with H3K4me1, H3K4me3, Pol II and H2A.Z, and no correlation with H3K27me3 (**Figure 1C** and **Figure S1C**).

All together, these data indicate that the absence of the U3 region of the LTR of the SIN-MLV vector causes no significant change in the integration preferences of MLV in the genome of human HSPCs.

### MLV and SIN-MLV vectors integrate in the same hot spots in the HSPC genome

MLV and SIN-MLV integrations showed the same non-random, highly clustered distribution in the human genome, with integration hot and cold spots. Integration clusters were statistically defined as described [4], obtaining a numerosity-adjusted threshold for cluster definition of 3 integrations within 31,525 bp for SIN-MLV and 12,587 bp for MLV at a *P*-value<0.01. We identified 1,415 clusters containing 56.0% (7,318) of the total SIN-MLV integration sites, an overall clustering highly comparable (*P*>0.1) to that showed by the MLV vector (3,497 clusters containing 65.3% of the integrations). Most of the SIN-MLV clusters (75.9%) overlapped for at least 1 bp with MLV clusters, and up to 69% overlapped for at least 1,000 bp. The non-overlapping clusters contained only 3 integrations and mapped less than 200-bp apart in the MLV and SIN-MLV datasets.

**Figure 2:**
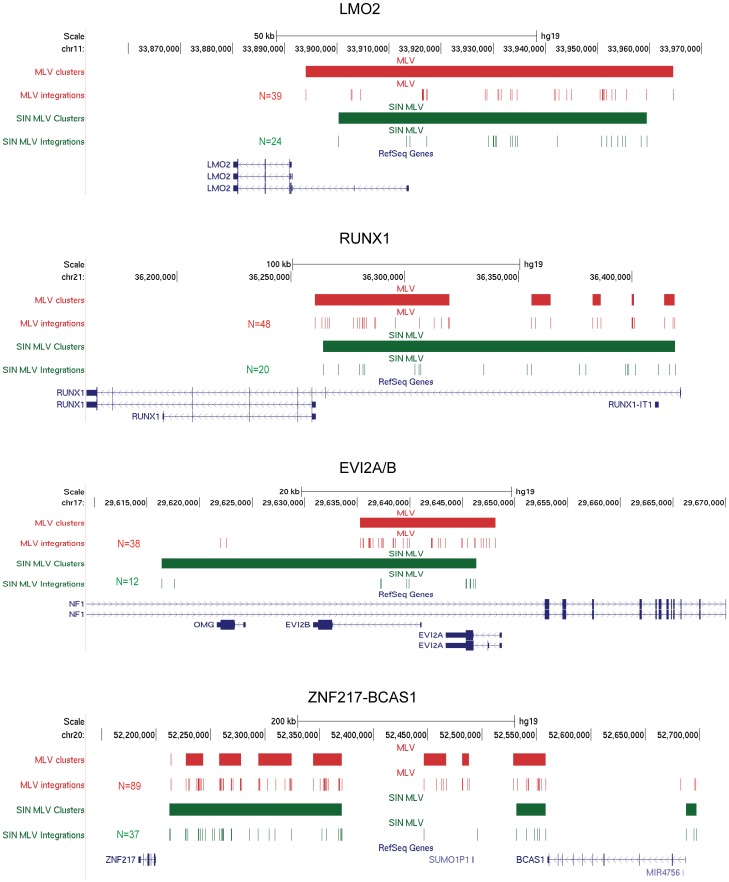
MLV and SIN-MLV integration sites and clusters in CD34^+^ HSPC-specific loci. Distribution of MLV (red) and SIN-MLV (green) integration clusters (horizontal solid bars) and integrations (vertical marks) in the LMO2, RUNX1, EVI2A/B, and ZNF217-BCAS1 loci as displayed by the UCSC Genome Browser. The base position feature at the top (scale bar and chromosome number) identifies the genomic coordinates of the displayed region.

We then looked at the integration clusters of both vectors in a number of individual loci characteristically hit at high frequency by MLV integration. Most of the loci highly targeted by MLV were targeted also by SIN-MLV. Whenever the numerosity was sufficient, we observed a striking overlap between the integration hot spots of the two vectors within the same loci. As examples, SIN-MLV integrations faithfully reproduced the MLV integration patterns in the LMO2, EVI2A/B, RUNX1, RUNX2, ZNF217-BCAS1, CD34, ELF1 and NFE2 loci, which were targeted at the same overall frequency and at the same hot spots within each locus (**Figure 2** and **Figure S2)**. We also found SIN-MLV integrations mapping very closely to the MLV integrations in two hot spots (MECOM and PRDM16) observed in the CGD and WAS clinical trials [13], [14], [15] (**Figure S2**).

## Discussion

Retroviruses select their target integration sites by tethering their PICs to the host cell chromatin through protein-protein interactions that appear to be specific for each retrovirus type [2]. The chromatin component LEDGF/p75 has a major role in tethering HIV PICs to the body of active transcription units, associated with H4K12ac, H2BK5me1, H3K27me1, H3K36me3, and H4K20me1 histone modifications [5], [25]. LEDGF/p75 is directly bound by the HIV integrase [26], a major viral determinant of target site selection [27]. Much less is known for the MLV PICs: regions preferred by MLV are marked by acetylations of histones H2A, H2B, H3 and H4, methylations of H3 (H3K4me1, H3K4me2, H3K4me3), and binding of Pol II, CTCF, histone acetyl transferases (p300 and CBP) and H2A.Z [4], [23], a histone variant enriched at targets of the Polycomb complex and marking elements involved in the regulation of cell commitment and differentiation [28]. Most of these associations are statistically significant for all MLV integrations, independently from their location with respect to promoters and in all analyzed cell types, possibly reflecting the engagements of PICs by basal components of the enhancer-binding and/or RNA Pol II transcriptional machinery. A yeast two-hybrid analysis of proteins potentially interacting with the MLV integrase provided biochemical evidence in this direction [29].

The peculiar characteristics of MLV integration, coupled with the strong transcriptional enhancer activity of the LTR U3 region, explain the high risk of insertional gene activation and genotoxicity observed in pre-clinical [19], [30], [31], [32] as well as clinical [11], [12], [13], [14], [15] studies. SIN vectors have been developed to reduce the genotoxic potential of MLV vectors, and have indeed shown a reduced cell immortalization and tumorigenic activity by sensitive pre-clinical assays [18], [19], [33]. In this study, we compared the integration preferences of a SIN and a traditional MLV vector in human CD34^+^ HSPCs, the most used target cell in clinical applications of retroviral transgenesis. By an LM-PCR coupled to pyrosequencing approach we show that the lack of the U3 enhancer/promoter in the LTR has no impact on the integration pattern of MLV in human HSPCs. Indeed, the SIN-MLV integration map reproduced with remarkable precision that of the unmodified MLV, including the association with TSSs, CpG islands, CNCs and representative epigenetic marks of active and highly regulated enhancers and promoters. SIN-MLV and MLV integrations cluster into hot spots at approximately the same frequency, and generate almost overlapping integration maps at the level of highly targeted loci, including the gene involved in most of the severe adverse events observed in clinical trials, i.e., LMO2 [11], [12]. On the basis of the integration pattern, a SIN-MLV vector therefore maintains the same genotoxic potential of a traditional MLV vector. Insertion of either provirus has a high chance of altering gene regulation by disrupting the physical continuity of enhancers and promoters, and by altering the chromatin configuration induced by the binding of transcription factors and the basal transcriptional machinery (the enhanceosome). This type of effect is not expected to differ between SIN-MLV and MLV vectors. On the contrary, the lack of the two copies of the U3 enhancer may significantly reduce the dominant activity in overcoming gene regulation typical of oncogenic retroviruses *in vivo* [19], although studies based on *in vitro* immortalization of bone marrow-derived cells provided conflicting evidence on this point [18], [34]. The choice of a cellular, possibly restricted enhancer to drive the internal transgene cassette may therefore overcome the LTR-specific component of the MLV genotoxicity. Indeed, most of the gene deregulation and genotoxic activity of SIN vectors appears to be due to the characteristics of the enhancer driving transgene expression more than by the SIN design *per se* [16], [17], [18], [19].

Genotoxicity of retroviral vectors has many components, including the vector design, the nature of the target cell and the genetic background of the patient, all ultimately affecting the risk of a specific gene therapy approach [35]. Target site selection is just one of these components. Based on current knowledge, SIN lentiviral vectors appear to combine an integration profile that does not target regulatory elements with the lack of strong viral enhancers. SIN-MLV vectors share with SIN lentiviral vectors only the latter component. On the other hand, the lower propensity to integrate within transcribed regions may reduce the recently emerged post-transcriptional component of insertional gene deregulation [23], [36], [37], [38], [39], [40]. For SIN-MLV vectors, the designs of the transgene expression cassette, and particularly the choice of its transcriptional regulatory elements, appear to be the most relevant determinants of their biosafety characteristics.

## Materials and Methods

### Vectors and cells

Human CD34^+^ HSPCs were purified form umbilical cord blood, pre-stimulated for 48 hours in serum-free Iscove modified Dulbecco medium supplemented with 20% FCS, 20 ng/ml human thrombopoietin, 100 ng/ml Flt-3 ligand, 20 ng/ml interleukin-6, and 100 ng/ml stem cell factor, as previously described [3]. HSPCs were transduced with the SIN-MLV vector pSRS11.EFS.GFP.pre, expressing GFP under the control of the elongation factor 1α promoter, pseudotyped in an amphotropic envelope by three-plasmid transfection in 293 cells, as previously described [16], [21]. Cells were infected by 3 rounds of spinoculation (1,500 rpm for 45 min) in the presence of 4 µg/ml polybrene. Transduction efficiency was evaluated by cytofluorimetric analysis of GFP expression 48 hrs after infection. All human studies were approved by the San Raffaele Scientific Institute Ethical Committee. Written informed consent was received from participants prior to inclusion in the study.

### Amplification, sequencing, and analysis of retroviral integration sites

Genomic DNA was extracted from a pool of 2×10^6^ CD34^+^/GFP^+^ cells enriched by fluorescence-activated cell sorting, after a brief period in culture to dilute unintegrated vectors. 3′-LTR vector-genome junctions were amplified by LM-PCR adapted to the GS-FLX Genome Sequencer (Roche/454 Life Sciences) pyrosequencing platform, as previously described [3], [4]. Raw sequence reads were processed by an automated bioinformatic pipeline that eliminated small and redundant sequences [4] and mapped on the UCSC hg19 release of the human genome. All UCSC Known Genes having their TSS at ±50 kb from an integration site were annotated as targets. Genomic features were annotated when their genomic coordinates overlapped for ≥1 nucleotide with a ±50-kb interval around each integration site. We used UCSC tracks for both cytosine-phosphate-guanosine (CpG) islands and conserved TFBSs. The genomic coordinates of 82,335 mammalian conserved non-coding sequences (CNCs) were previously described [22]. For the association of the integrations with epigenetic marks, we re-annotated published ChIP-Seq data [24] in the UCSC hg19 release of the human genome, and analyzed the distribution of histone modifications (H3K4me1, H3K4me3, H3K27me3) H2A.Z and Pol II binding sites around the integrations, using the seqMINER platform [41]. Previously generated MLV integrations and random control sequences datasets [4] were also re-annotated on the UCSC hg19 genome. For all pairwise comparisons we applied a 2-sided Fisher's exact test. The threshold for statistical significance was set at a *P* value<0.05.

## Supporting Information

Figure S1 Association between MLV and SIN-MLV integration sites and CpG islands, CNCs, PolII and histone modifications. Distribution of the distance of SIN-MLV (green bars) and MLV wt (red bars) integrations from the midpoint of CpG islands (A) or CNCs (B) in a 20 kb window. In the y axis is plotted the percentage of the total number of CpG islands or CNCs located at ±50 kb distance from the integrations. The black line indicates the distribution of random control sites. (C) The distribution of epigenetic marks in a 10 kb window around vector integration sites (IS) shown for H3K27me3 (top panels), H2A.Z (middle panels), PolII (lower panels) with respect to MLV integrations (left panels) or SIN-MLV integrations (right panels). See legend of Figure 1C for explanation of the graphs.
(PDF)

Figure S2 MLV and SIN-MLV integration sites and clusters in CD34^+^ HSPC-specific loci. Distribution of MLV (red) and SIN-MLV (green) integration clusters (horizontal solid bars) and integrations (vertical marks) in the CD34, ELF1, NFE2, and RUNX2, MECOM and PRDM16 loci as displayed by the UCSC Genome Browser. The base position feature at the top (scale bar and chromosome number) identifies the genomic coordinates of the displayed region.
(PDF)
