# Supplementary material for: Deletion of the LTR Enhancer/Promoter Has No Impact on the Integration Profile of MLV Vectors in Human Hematopoietic Progenitors
Source: PLoS One. 2013 Jan 31;8(1):e55721. doi: 10.1371/journal.pone.0055721 (PMC3561312; doi:10.1371/journal.pone.0055721)
Supplement: Figure S1 — Association between MLV and SIN-MLV integration sites and CpG islands, CNCs, PolII and histone modifications. Distribution of the distance of SIN-MLV (green bars) and MLV wt (red bars) integrations from the midpoint of CpG islands (A) or CNCs (B) in a 20 kb window. In the y axis is plotted the percentage of the total number of CpG islands or CNCs located at ±50 kb distance from the integrations. The black line indicates the distribution of random control sites. (C) The distribution of epigenetic marks in a 10 kb window around vector integration sites (IS) shown for H3K27me3 (top panels), H2A.Z (middle panels), PolII (lower panels) with respect to MLV integrations (left panels) or SIN-MLV integrations (right panels). See legend of Figure 1C for explanation of the graphs. (PDF) [file pone.0055721.s001.pdf]

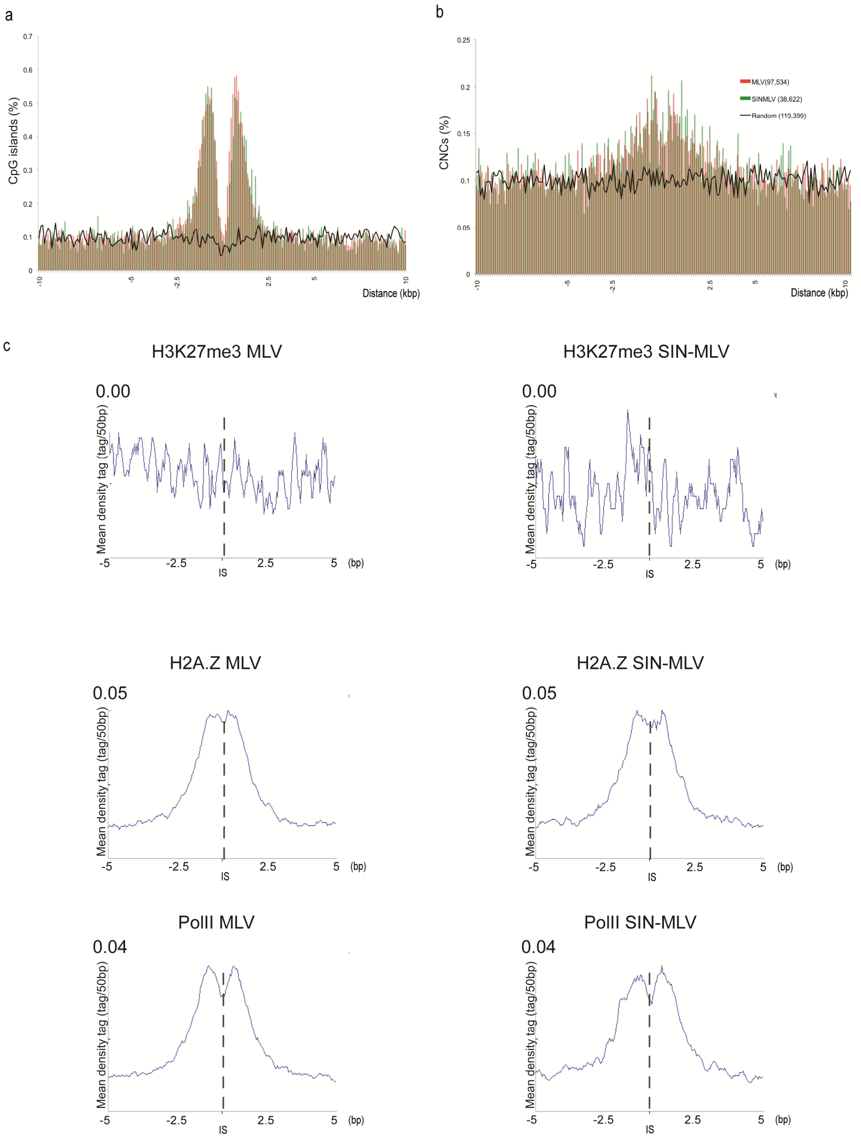

Figure S1. Association between MLV and SIN-MLV integration sites and CpG islands, CNCs, PolII and histone modifications. Distribution of the distance of SIN-MLV (green bars) and MLV wt (red bars) integrations from the midpoint of CpG islands (A) or CNCs (B) in a 20kb window. In the y axis is plotted the percentage of the total number of CpG islands or CNCs located at ±50kb distance from the integrations. The black line indicates the distribution of random control sites. (C) The distribution of epigenetic marks in a 10kb window around vector integration sites (IS) is shown for H3K27me3 (top panels), H2A.Z (middle panels), PolII (lower panels) with respect to MLV integrations (left panels) or SIN-MLV integrations (right panels). See legend of Figure 1C for explanation of the graphs.
