# Supplementary material for: Deletion of the LTR Enhancer/Promoter Has No Impact on the Integration Profile of MLV Vectors in Human Hematopoietic Progenitors
Source: PLoS One. 2013 Jan 31;8(1):e55721. doi: 10.1371/journal.pone.0055721 (PMC3561312; doi:10.1371/journal.pone.0055721)
Supplement: Figure S2 — MLV and SIN-MLV integration sites and clusters in CD34+ HSPC-specific loci. Distribution of MLV (red) and SIN-MLV (green) integration clusters (horizontal solid bars) and integrations (vertical marks) in the CD34, ELF1, NFE2, and RUNX2, MECOM and PRDM16 loci as displayed by the UCSC Genome Browser. The base position feature at the top (scale bar and chromosome number) identifies the genomic coordinates of the displayed region. (PDF) [file pone.0055721.s002.pdf]

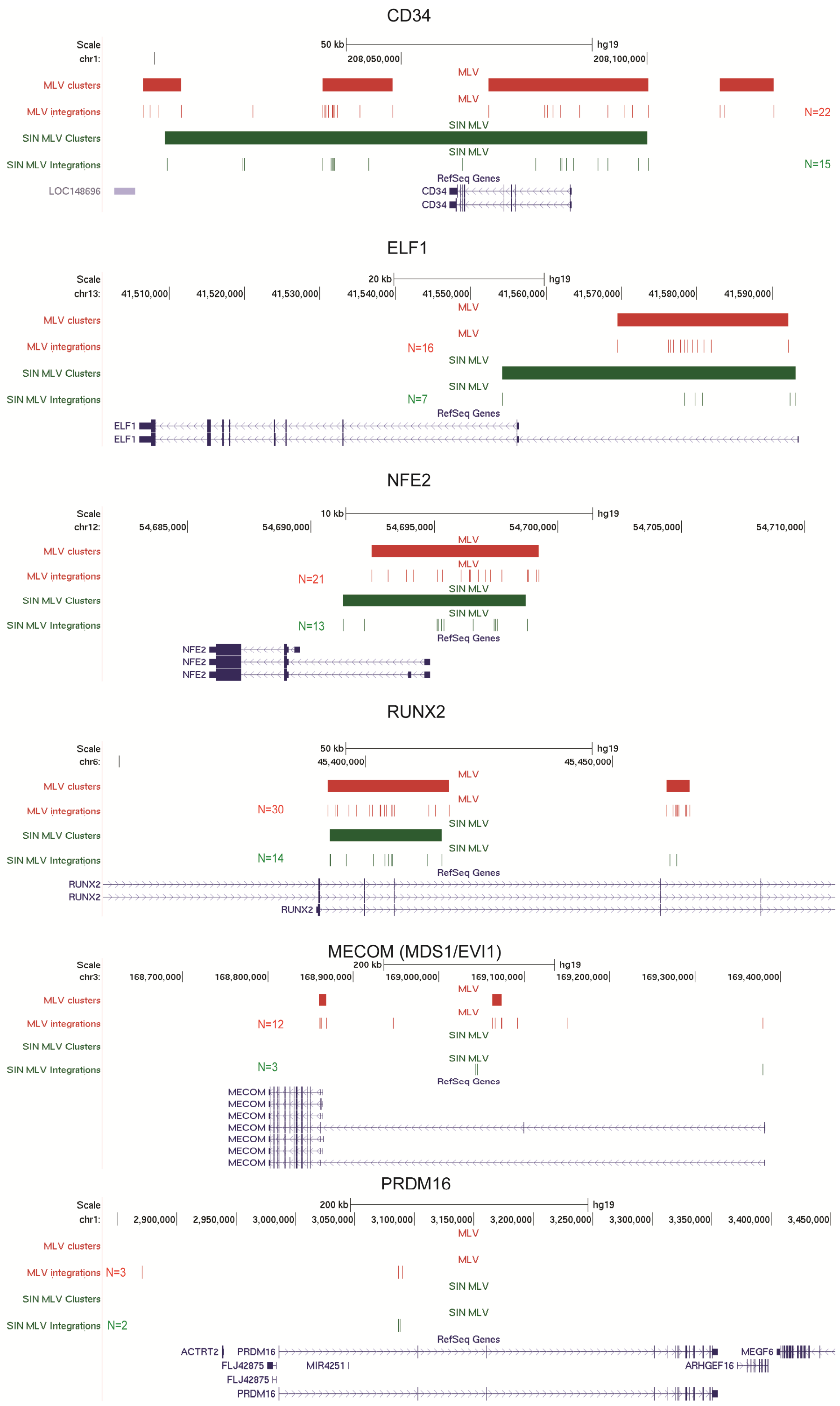

Figure S2. MLV and SIN-MLV integration sites and clusters in CD34+ HPC-specific loci. Distribution of MLV (red) and SIN-MLV (green) integration clusters (horizontal solid bars) and integrations (vertical marks) in the CD34, ELF1, NFE2, and RUNX2, MECOM and PRDM16 loci as displayed by the UCSC Genome Browser. The base position feature at the top (scale bar and chromosome number) identifies the genomic coordinates of the displayed region.
